# Google Trends for Pain Search Terms in the World’s Most Populated Regions Before and After the First Recorded COVID-19 Case: Infodemiological Study

**DOI:** 10.2196/27214

**Published:** 2021-04-22

**Authors:** Istvan-Szilard Szilagyi, Torsten Ullrich, Kordula Lang-Illievich, Christoph Klivinyi, Gregor Alexander Schittek, Holger Simonis, Helmar Bornemann-Cimenti

**Affiliations:** 1 Department of Anesthesiology and Intensive Care Medicine Medical University of Graz Graz Austria; 2 Institute of Computer Graphics and Knowledge Visualisation Graz University of Technology Graz Austria; 3 Fraunhofer Austria Research GmbH Graz Austria; 4 Department of Anesthesiology, Perioperative and Intensice Care Medicine University Hospital Salzburg Salzburg Austria

**Keywords:** COVID-19, data mining, Google Trends, incidence, internet, interest, pain, research, trend

## Abstract

**Background:**

Web-based analysis of search queries has become a very useful method in various academic fields for understanding timely and regional differences in the public interest in certain terms and concepts. Particularly in health and medical research, Google Trends has been increasingly used over the last decade.

**Objective:**

This study aimed to assess the search activity of pain-related parameters on Google Trends from among the most populated regions worldwide over a 3-year period from before the report of the first confirmed COVID-19 cases in these regions (January 2018) until December 2020.

**Methods:**

Search terms from the following regions were used for the analysis: India, China, Europe, the United States, Brazil, Pakistan, and Indonesia. In total, 24 expressions of pain location were assessed. Search terms were extracted using the local language of the respective country. Python scripts were used for data mining. All statistical calculations were performed through exploratory data analysis and nonparametric Mann–Whitney *U* tests.

**Results:**

Although the overall search activity for pain-related terms increased, apart from pain entities such as headache, chest pain, and sore throat, we observed discordant search activity. Among the most populous regions, pain-related search parameters for shoulder, abdominal, and chest pain, headache, and toothache differed significantly before and after the first officially confirmed COVID-19 cases (for all, *P*<.001). In addition, we observed a heterogenous, marked increase or reduction in pain-related search parameters among the most populated regions.

**Conclusions:**

As internet searches are a surrogate for public interest, we assume that our data are indicative of an increased incidence of pain after the onset of the COVID-19 pandemic. However, as these increased incidences vary across geographical and anatomical locations, our findings could potentially facilitate the development of specific strategies to support the most affected groups.

## Introduction

Eysenbach [1] in 2002 defined Infodemiology as the “science of distribution and determinants of information in an electronic medium, specifically the Internet, or in a population, with the ultimate aim to inform public health and public policy.” Since then, web-based sources have been suggested to be valuable in various academic fields for understanding timely and regional differences in the public interest in certain terms and concepts [2].

Especially in health and medical research, infodemiological studies using Google Trends have increased over the last decade [3]. Recently, several studies have used Google Trends data to elucidate the progression of the COVID-19 pandemic. For example, Husain et al [4] used Google Trends to track public interest in COVID-19 [4] and reported that in the United States, when containment and mitigation strategies were first implemented, public interest was very low. However, the search volume of COVID-19–related terms increased and correlated with the increase in positive findings on COVID-19 tests nationwide. Furthermore, Walker et al [5] correlated the search activity for the term “loss-of-smell,” an early symptom of COVID-19, to the number of daily confirmed cases and COVID-19 deaths. Similarly, Jimenez et al [6] correlated searches for different symptoms of COVID-19 with daily incidences. Another study used Google Trends to identify mental health consequences related to physical distancing during the lockdown. Notably, Knipe et al [7] reported a reduction in search activity related to suicide and depression after the announcement of the pandemic.

The use of internet searches to gather information about patients experiencing pain has been insufficiently studied. Of 2 studies on populations of people with chronic pain, 1 reported that 24% of the population searched the internet for pain-related information, and the other reported that 39% of the population performed internet searches [8,9]. However, both studies are more than 10 years old. Considering the recent growth of the usage of online media, it is reasonable to suppose that the proportion of individuals searching for pain-related information on the internet increased during the last decade.

The COVID-19 pandemic has affected the health condition and quality of life of people with chronic pain in 2 ways. First, several changes have led to increased susceptibility to and a higher risk of pain exacerbation. Specifically, the availability of health care was significantly reduced and an atmosphere of fear and isolation was developed [10]. Previous experiences with epidemics have shown a latent, but long-term, deterioration of psychosocial problems [11]. Therefore, it has also been postulated that chronic pain, as a biopsychosocial phenomenon, exacerbates in response to the COVID-19 situation [12]. Second, acute stress can alleviate chronic pain [13,14]. Indeed, acute stress in response to disasters alleviates pain [15]. One could therefore hypothesize that the COVID-19 situation alleviates chronic pain, at least at the onset of the pandemic. To date, no studies have examined changes in the experience of chronic pain after the onset of the COVID-19 pandemic in large patient populations. To bridge this knowledge gap, search activity can be used as a surrogate for the public interest in pain. This does not necessarily reflect the incidence or the burden of pain in the general population; however, acute changes can be interpreted as a momentary shift of attention and thus highlight the importance of these symptoms.

This study aimed to assess the Google search activity of pain-related expressions in the most populated regions worldwide and to use them as markers of the social importance of pain before and during the first wave of the COVID-19 pandemic.

## Methods

**Figure 1:**
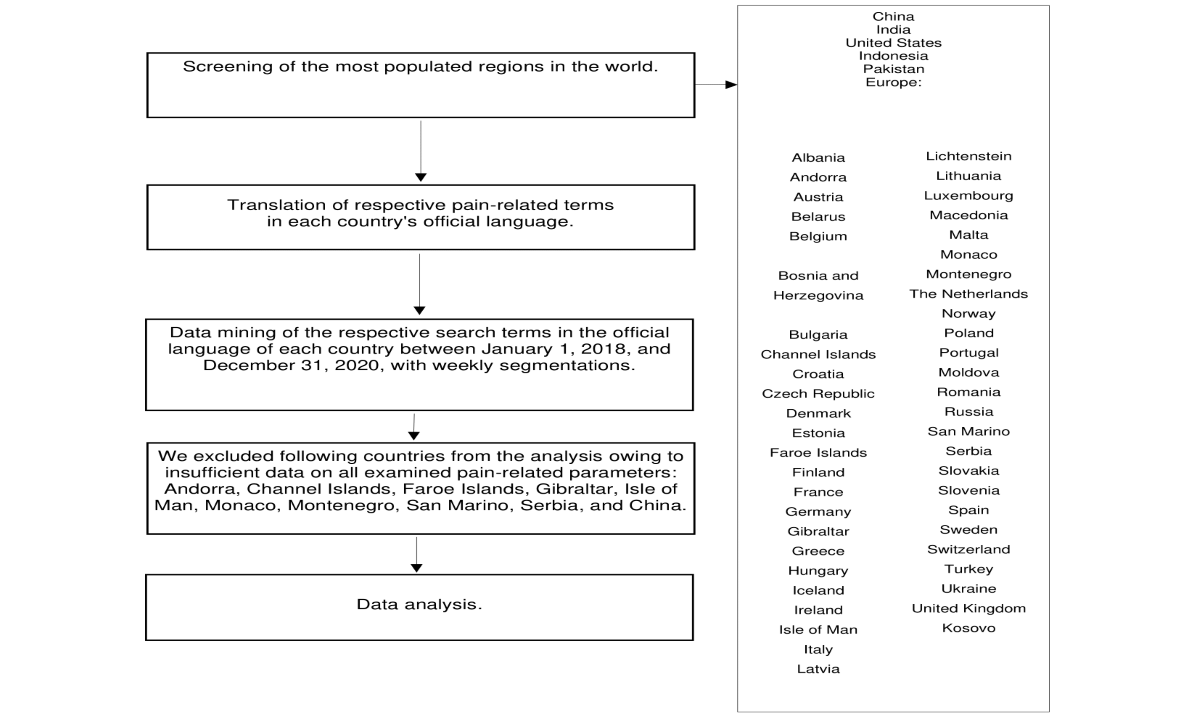
Workflow for data retrieval and processing pipeline in this study.

This infodemiological study was designed and carried out at Graz Medical University in cooperation with the Graz University of Technology and Fraunhofer Austria. The workflow of this study is presented in Figure 1. The temporary popularity of pain-related search terms was assessed using Google Trends [16]. Google Trends delivers data on web-based search queries for specified timeframes and countries. Google Trends offers 2 analysis modes: one with “terms” and the other with “topics.” In contrast to the analysis with “terms,” Google Trends analysis with “topics” corresponds to a fuzzy search. The difference is that “topics” include all search terms related to a particular aspect, whereas “terms” are specific, and the results will only show the relative volume of the corresponding term. Since different types of pain are related to one another—for example, neck pain and shoulder pain or dysmenorrhea and pelvic pain—a fuzzy search that includes related terms is not adequately discriminative. Furthermore, it is unclear which terms are summarized in a specific “topic.” Hence, smaller, discriminative trend analysis with “terms” was preferred. Data are presented as relative popularity, normalized to the region and period and scaled from 0 to 100. Subsequently, the data were exported for further calculations [17]. The study followed the methodology framework developed by Mavragani and Ochoa [18].

The search terms used in this study were based on a recent study of global internet searches associated with pain [19]. In total, 24 expression entities based on anatomical regions were used: “abdominal pain,” “back pain,” “breast pain,” “chest pain,” “dysmenorrhea,” “dyspareunia,” “ear pain,” “epigastric pain,” “eye pain,” “groin pain,” “headache,” “knee pain,” “low back pain,” “neck pain,” “odynophagia,” “pelvic pain,” “penile pain,” “podalgia,” “rectal pain,” “shoulder pain,” “sore throat,” “testicular pain,” “toothache,” and “wrist pain.” The results for 3 of these terms (“penile pain,” “podalgia,” and “rectal pain”) were excluded from further analysis because of insufficient data. Weekly data sets were downloaded from January 1, 2018, to December 31, 2020. Google Trends analysis was carried out in February 2021.

Our intention was to provide an overview of the development of the aforementioned pain-related search parameters in the most populated regions worldwide. The most populous areas worldwide were extracted from the recommendations of the United Nations and a web-based population database [20,21]. Europe, consisting of individual European countries, is defined as a single region in this study. Figure 1 shows all the selected regions and the individual European countries. We identified the following as the most populated regions: Europe, the United States, China, India, Pakistan, Brazil, and Indonesia. Search terms were extracted in the respective language of each country. The data set of Europe is accumulated; it consists of all available nationwide data sets of the European countries weighted by their population. Details including the complete list of countries, their population sizes, the corresponding weighting factors, and the date of the first officially reported COVID-19 case of the country were provided by the European Centre for Disease Prevention and Control. Using this list of European countries, we retrieved a data vector for each country and pain type in the respective language of each country. Thereafter, the individual vectors were weighted with the country’s population and accumulated to a European vector (Multimedia Appendix 1). Since the weighted summation with sum 1 is an affine map, the result is also normalized. Calculations with the European vector thus correspond to stratified sampling; that is, individual characteristics of the target group deemed relevant for the study are transferred to the sample in approximately the same proportion as those in the population.

Data from China could not be extracted adequately, and data extraction yielded numerous error parameters. Therefore, China had to be excluded from our analysis.

We assumed that the occurrence of biological and psychosocial effects of the pandemic correlated with the first appearance of the virus in a certain region. Accordingly, we defined the beginning of the “COVID-19 period” for each geographic region with the first officially confirmed case for each country on the basis of reports from the European Centre for Disease Prevention and Control [22]. The date of the first officially reported COVID-19 case for each country was used as the independent variable for our calculations.

For data extraction, we used Python scripts. The Google Trends analysis data were obtained with the software libraries pytrends and pandas [23,24] using Python. The number of COVID-19 cases were obtained from the European Centre for Disease Prevention and Control on February 9, 2021. Results from countries with erroneous data or insufficient cases and subsequently inconsistent data are excluded from the study (Figure 1).

IBM SPSS Statistics (version 26, IBM Corp), Microsoft Office 365, and LibreOffice 7.1 were used for the statistical calculations. Explorative data analysis and nonparametric Mann–Whitney *U* tests were performed. The level of significance was not corrected for multiple comparisons. These corrections are necessary for multiple tests carried out with the same samples: the greater the number of hypotheses tested on 1 data set, the higher the probability that 1 of them is (incorrectly) assumed to be true. As the data were collected separately by country and pain type, the precondition for test corrections (all tests on 1 data set) was not met; that is, Bonferroni corrections or similar methods were not required and hence not applied.

**Figure 2:**
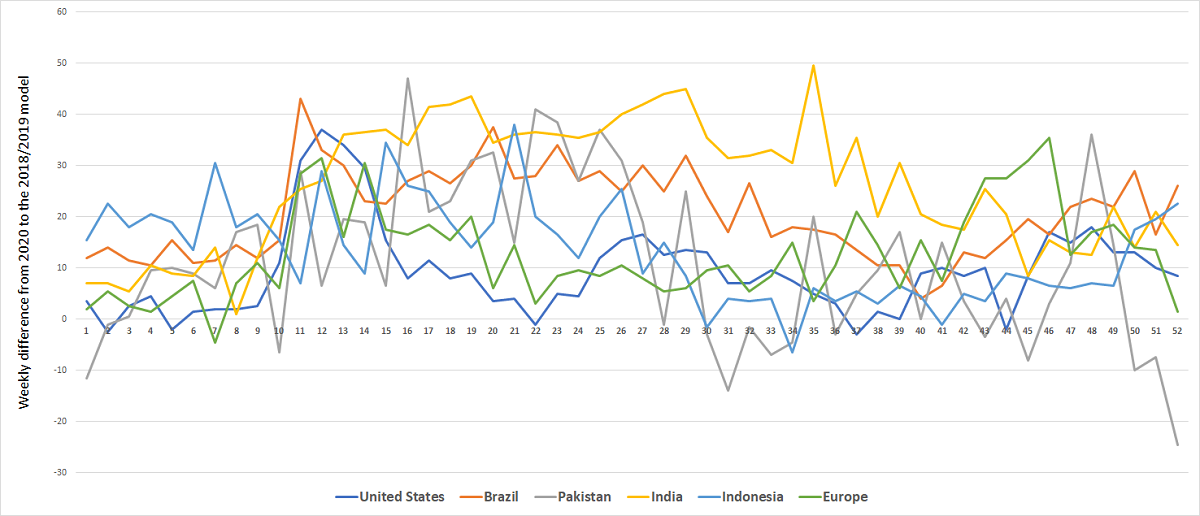
Trends for headache.

**Figure 3:**
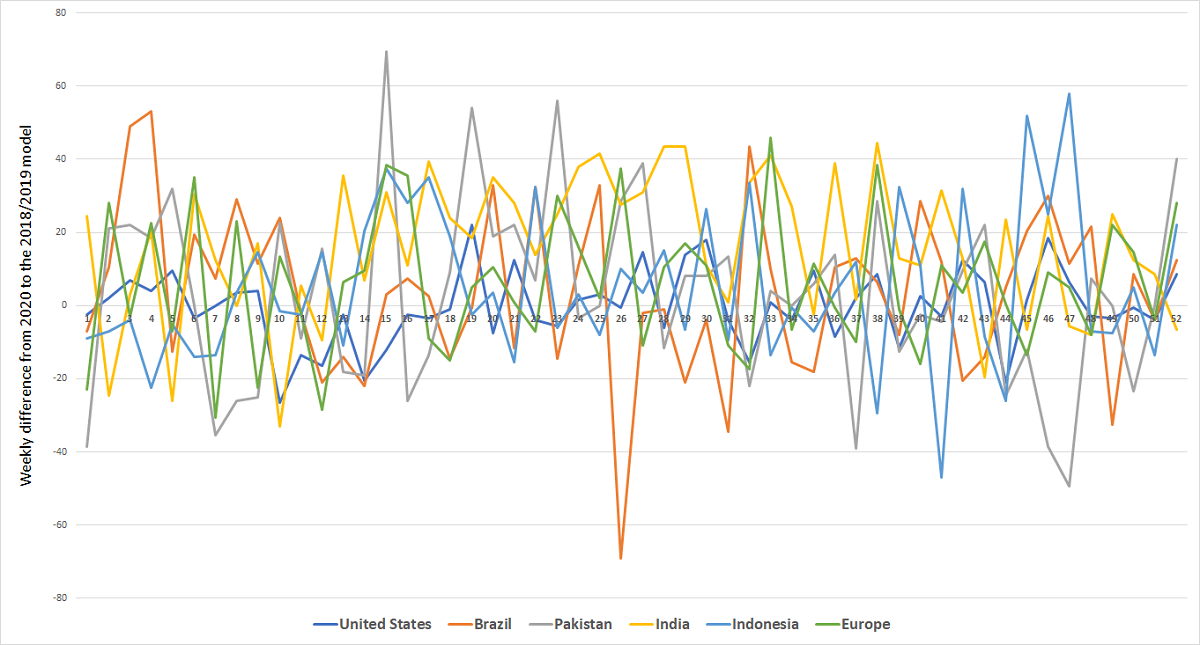
Trends for wrist pain.

**Figure 4:**
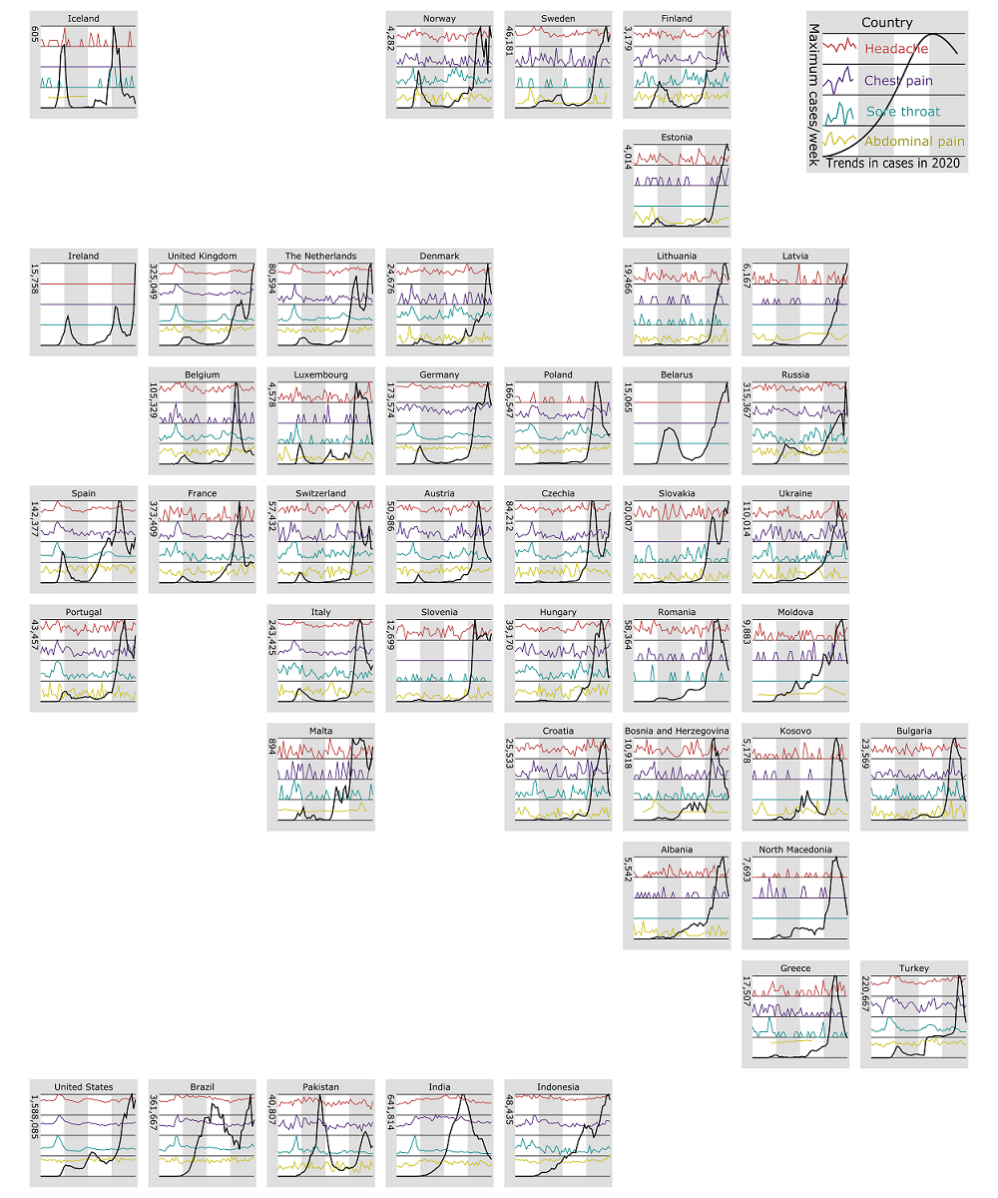
An overview of the 5 most populous countries (last row) and European countries in an arrangement that corresponds to the geography of Europe. Only countries for which sufficient data were available are included; data on China and Serbia were inadequate. Each chart consists of 4 rows, each used by a subchart, and 4 vertical columns, each covering 1 quarter of 2020.

Additionally, we performed a visual analysis to illustrate the difference between significant and nonsignificant changes in the time series data (Figures 2 and 3) and to plot a side-by-side comparison of the progression of pain-related search criteria with the so-called waves of COVID-19 outbreaks (Figure 4). This analysis has been performed for “headache,” as this query term increased significantly in all geographic regions, and for “wrist pain,” as this query term did not show any significant changes.

For both terms, the analysis involved a seasonal autoregressive model whose parameters have been determined using Google Trends vectors retrieved for 2018 and 2019. Using these models, weekly differences between the model values for 2018-2019 and 2020 were calculated and plotted (Figures 2 and 4).

## Results

**Figure 5:**
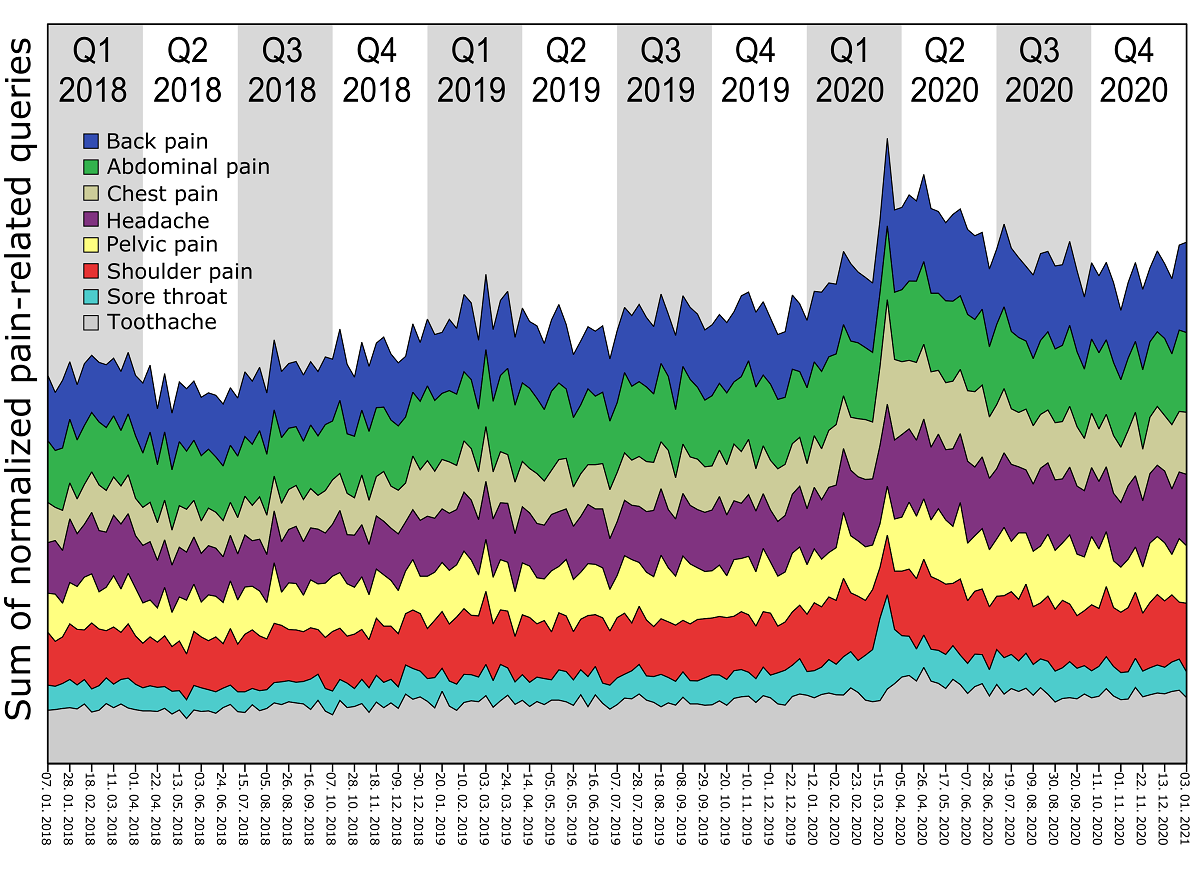
Summation trends of pain-related search parameters in the most populated regions worldwide (Europe, the United States, Brazil, Pakistan, India, and Indonesia).

Our results demonstrate significant differences in pain-related search queries by comparing quantities before and after the first confirmed COVID-19 case. We observed a peak in the incidence of pain-related search parameters in March and April 2020 (Figure 5). Abdominal pain, dysmenorrhea, dyspareunia, groin pain, eye pain, knee pain, low back pain, and pelvic pain were the only pain types with a significantly decreased frequency of search relevance in some of the most populous geographic regions since the COVID-19 outbreak (Table 1). In contrast, the frequency of searches related to back pain, breast pain, chest pain, ear pain, headache, odynophagia, neck pain, shoulder pain, sore throat, testicular pain, toothache, and wrist pain significantly increased after the first confirmed case of COVID-19 was reported (Table 1, Figure 4).

**Table 1:** Comparison of search trends between January 1, 2018, and December 31, 2020, for pain-related terms before and after the first officially confirmed COVID-19 case in in Europe, the United States, Brazil, Pakistan, India, and Indonesia (China was excluded from the data set owing to insufficient data).

Pain type	Europe (*P* value)	United States (*P* value)	Brazil (*P* value)	Pakistan (*P* value)	India (*P* value)	Indonesia (*P* value)	Overall (*P* value)
Back pain	<.001^a^	.09 ^b^	<.001^a^	.04^a^	<.001^a^	<.001^a^	<.001^a^
Abdominal pain	<.001^a^	<.001^c^	<.001^a^	.05^a^	<.001^a^	<.001^a^	<.001^a^
Breast pain	<.001^a^	.07 ^b^	<.001^a^	.10 ^b^	<.001^a^	<.001^a^	<.001^a^
Chest pain	<.001^a^	<.001^a^	<.001^a^	<.001^a^	<.001^a^	<.001^a^	<.001^a^
Dysmenorrhea	.15 ^b^	.35 ^b^	.09 ^b^	.13 ^b^	<.001^a^	.009^c^	.12 ^b^
Dyspareunia	.12 ^b^	<.001^c^	.31 ^b^	.22 ^b^	.20 ^b^	.86^b^	.28 ^b^
Ear pain	.01^a^	.84> ^b^	.06 ^b^	.01^a^	<.001^a^	.003^a^	<.001^a^
Epigastric pain	.45 ^b^	.61 ^b^	<.001^a^	.31 ^b^	.11 ^b^	N/A^d^	<.001^a^
Groin pain	.64 ^b^	<.001^c^	<.010^a^	.84 ^b^	<.001^a^	.03^c^	.73 ^b^
Eye pain	<.001^a^	.47 ^b^	<.001^a^	.02^a^	.05^a^	.03^c^	<.001^a^
Headache	<.001^a^	<.001^a^	<.001^a^	.03^a^	<.001^a^	<.001^a^	<.001^a^
Knee pain	.43 ^b^	<.001^c^	.42 ^b^	.23 ^b^	.04^a^	<.001^a^	.09 ^b^
Low back pain	.01^a^	<.001^c^	<.001^a^	.06 ^b^	.23 ^b^	<.001^c^	.77 ^b^
Odynophagia	<.001^a^	.45 ^b^	<.001^a^	.05 ^b^	.22 ^b^	.40 ^b^	.01^a^
Neck pain	<.001^a^	.44 ^b^	.03^a^	.08 ^b^	<.001^a^	<.001^a^	<.001^a^
Pelvic pain	.01^a^	<.001^c^	<.001^a^	.03^a^	<.001^a^	<.001^a^	<.001^a^
Shoulder pain	.01^a^	.40 ^b^	<.001^a^	<.001^a^	<.001^a^	<.001^a^	<.001^a^
Sore throat	<.001^a^	.05^a^	<.001^a^	<.001^a^	<.001^a^	.23 ^b^	<.001^a^
Testicular pain	.42 ^b^	.16 ^b^	.10 ^b^	.45 ^b^	<.001^a^	<.001^a^	<.001^a^
Toothache	<.001^a^	.31 ^b^	<.001^a^	<.001^a^	<.001^a^	<.001^a^	<.001^a^
Wrist pain	.08 ^b^	.94 ^b^	.11 ^b^	.77 ^b^	<.001^a^	.04^a^	<.001^a^

^a^Relevance of the pain-related search parameter increased significantly after the first confirmed COVID-19 case was reported.

^b^No significant changes.

^c^Relevance of pain-related search parameter decreased significantly after the first confirmed COVID-19 case was reported.

^d^N/A: not applicable; no data available/inadequate number of cases.

Our data show that in Europe, Brazil, Pakistan, and India, all pain entities listed above increased significantly or remained unchanged after the first confirmed COVID-19 case was reported. In contrast, in the United States, we observed an inhomogeneous trend, with the frequency of some pain-related search terms decreasing significantly after the first confirmed COVID-19 case was reported, whereas that of the others increased or remained unchanged (Table 1).

**Figure 6:**
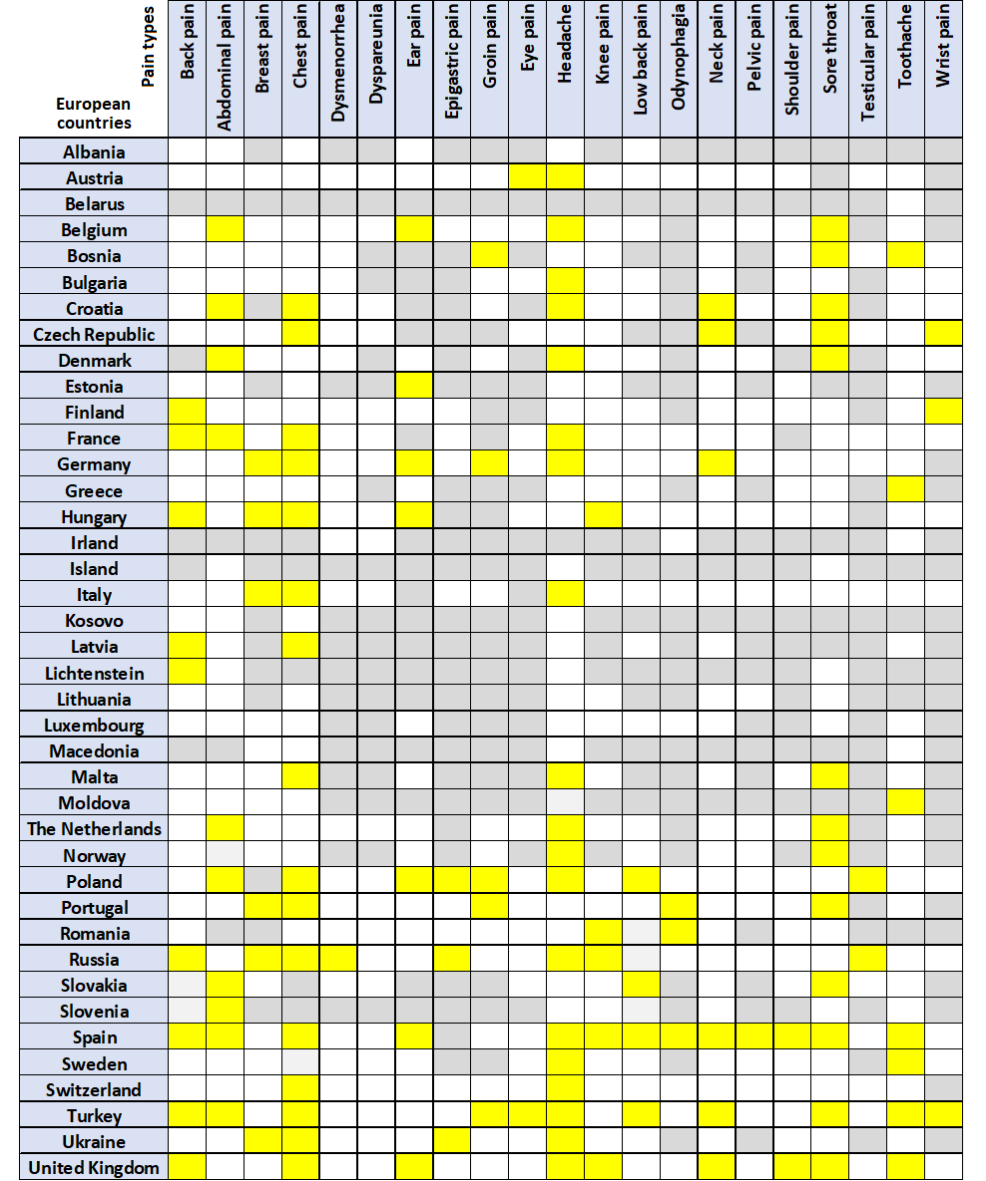
Summation trends of pain-related search parameters in European countries. Yellow=relevance of pain-related search parameters changed significantly after the report of the first confirmed COVID-19 case, white=no significant changes, and gray=no data available or inadequate data.

A comparison among individual European countries revealed that the most frequently observed significant differences among countries were noted for headache, chest pain, sore throat, abdominal pain, and back pain since the COVID-19 outbreak. With respect to individual European countries, Spain displayed the most frequent significant pre– and post–COVID-19 discrepancies in the frequency of pain-related search queries (Figures 3 and 6).

The analysis of the difference plots for the term “headache” revealed that the weekly differences between the 2020 and the 2018-2019 models were more often significantly positive than negative; in other words, the number of queries for “headache” increased. As the Google Trends data are normalized with a range of 0 to 100, the 2018-2019 model was normalized accordingly (Figure 2).

If the search frequency of a term did not increase significantly, the differences would be expected to be as often positive as negative. Consequently, the difference plots would resemble random noise; for example, the search term “wrist pain” displayed no significant change in most geographic regions and its plots illustrate random noise around 0.

Furthermore, on assessing country-specific developments in pain-related search parameters, which have significantly increased in relevance since the COVID-19 pandemic outbreak, it becomes apparent that the relevance of pain-related search parameters increased in almost all countries before the peak of the first wave of the COVID-19 pandemic. “Headache” was consistently high in relevance, especially in Belgium, Brazil, Germany, Indonesia, Italy, Russia, Turkey, and the United States. The relevance of “chest pain” and “sore throat” seemed to resonate with the first wave of the COVID-19 pandemic, as seen in several countries including Belgium, Croatia, France, Italy, the Netherlands, and the United Kingdom. In contrast, the frequency of “abdominal pain” appeared highly dynamic compared to that of COVID-19 cases (Figure 4).

## Discussion

### Principal Findings

Our results indicate that for most pain entities, the frequency of pain-related search queries significantly increased after the official report of the first confirmed COVID-19 case (Table 1). In particular, search queries for “chest pain” and “headache” significantly increased in all of the world's most populous regions included in this study.

On assessing the trends for the most populated regions, we found that the incidence of pain-related search criteria increased for back pain, breast pain, chest pain, ear pain, headache, neck pain, shoulder pain, sore throat, and toothache after the COVID-19 outbreak. With the exception of the United States and Indonesia, we observed a significant increase in pain-related search parameters for almost all the pain types (Table 1).

Part of our findings of increased numbers of searches of pain-related terms may reflect symptoms attributed to COVID-19. For example, sore throat [25] and headache [26] are frequent symptoms of COVID-19 and have been often discussed as signs of COVID-19 in the news media [27]. Consequently, an increased interest in this search term was expected. This may also apply to chest pain. Similar to sore throats and headaches, “chest tightness” was a term that was frequently used in the news media in connection with COVID-19 [27]. However, since chest pain is also a key symptom of major cardiac events, our data should be interpreted with caution as other studies have reported a drastic increase in heart attacks and cardiovascular deaths during the first wave of the COVID-19 pandemic [28]. This is postulated to be associated with delays in seeking help. Compared with other pain-related terms, the increase in searches for “toothache” was delayed but persisted at a higher level. The changes in search behavior for toothache may be explained by the fact that many patients had no opportunity or were too afraid to visit a dentist during the lockdown [29]. The number of dental visits decreased to less than one-fifth that before the onset of the COVID-19 pandemic [30]. Therefore, painful dental conditions may have remained untreated or may have developed over time. The variation in the search pattern of eye pain in response to the COVID-19 pandemic has been previously reported [31]. However, it remains unclear if this can be attributed to a direct effect of the virus or is the effect of changes in lifestyle during the lockdown (eg, more time spent in closed rooms and increased screen time). The same argument could hold true for neck pain; as internet usage for private purposes, remote working, and education increased, one can assume that problems with posture also became more prevalent [32].

It remains unclear why the search frequency for groin and knee pain decreased in certain populous regions before to after the onset of the COVID-19 pandemic. Each of these pain entities was associated with physical activity [33,34]. Owing to the potential consequences of a lockdown, decreased physical activity can be anticipated as a possible effect.

In Pakistan, India, Brazil, and Indonesia, the frequency of most pain-related keywords increased after the first confirmed COVID-19 case. Furthermore, Europe displayed a predominant increase in search queries across almost all pain types. On further inspection of individual European countries, we found that similar to the overall picture, that COVID-19–related symptoms dominate the increase in searches, such as “headache,” “sore throat,” or “chest pain” (Figure 6). On the contrary, in the United States the frequencies for most pain-related searches decreased.

Owing to our study design, we can only speculate the reasons for this geographically heterogeneous pattern. We assume that except for those symptoms (chest pain, headache, neck pain, sore throat, and toothache) that showed the same direction of change in all geographic regions, there are no direct or indirect biological effects of COVID-19 on pain. Thus, the reasons for the observed regional differences are most likely attributed to psychosocial dimensions. Differences in social systems and cultures are evident in various fields of health [35]. Especially in the more developed regions, the overall satisfaction with the health care system is generally high [36,37]. One could speculate that these differences contribute to the impact of the COVID-19 pandemic on people with chronic pain on an individual level.

A special situation was observed in the United States, in that we observed a reduction in the frequency of most search terms. This may be explained by the fact that the exposure to COVID-19 was time-shifted for different areas in the United States. Only the third wave of the pandemic encompassed the country simultaneously and its entirety.

### Limitations

This study has limitations, which need to be addressed. Some of these limitations are related to our study design. It is evident that analyzing online searches may only reflect the behavior of internet users, thus potentially excluding relevant groups; for example, older or uneducated individuals [38]. Other demographic groups are potentially overrepresented. Younger individuals and women displayed a higher prevalence in health-related information searches on the internet [39].

Similarly, one can only speculate the reasons for the individual searches. Generally, internet searches are considered a surrogate for public interest. This does not necessarily imply that searches are carried out by patients experiencing a certain symptom. In our case, relatives, advocates, or researchers may also search for pain-related terms.

Figure 4 shows high fluctuations in the Google Trends data set. According to Google, only a sample is used for the trend analysis and not the complete data set of all search queries [40]. Google does not disclose which sampling strategy is used. However, Figure 4 suggests that the population size has an influence on the sampling rate considering the number of search queries. The lower the sampling rate (ie, lesser data on which the data calculation is based), the greater the inaccuracies that are reflected in the fluctuations in the graphs [41].

Some limitations are specific to our study. For instance, our study only uses Google data and therefore represents the search behavior of only Google users. However, as Google has a nearly 70% worldwide market share, it is representative of the majority of internet users [42]. In our study, we excluded China as we did not obtain adequate data from this origin. The use of other sources including Baidu could have provided more details; however, as these data would not be comparable to Google Trends data, we decided against this approach.

We have summarized the data of European countries. In doing so, the exact timepoint of the “first official case” was not defined at the national level but rather on the continental level in in Europe. It can be assumed that the inaccuracy of these statistics for the temporal delimitation for the local onset of the pandemic is of the same order of magnitude as for the individual country data sets provided by Google Trends. The problem also exists in these data sets as outbreaks of the pandemic did not occur homogenously (eg, in all US states or in all provinces of China) simultaneously.

The definition of search terms is crucial when extracting information from search databases [38]. Our keywords represent anatomical locations instead of certain diagnoses (eg, fibromyalgia and migraine). This approach was previously introduced by Kamiński et al [19]. As we have not been aware of another already established search strategy for pain-related keywords in an infodemiological study, we decided to follow the method of Kamiński et al.

### Conclusions

Our results indicate considerable changes in internet search behavior related to pain-related keywords for the most populated regions worldwide. There are many possible reasons for these changes in internet search behavior. As expected, we found that certain search terms that are closely related to COVID-19 symptoms are increasing, such as the pain entities of headache, chest pain, or sore throat. Our study describes the analysis of the trends in pain-related search parameters on the Google search network, as developed over a 2-year period.

In summary, apart from COVID-19–related pain symptoms, the frequency of search parameters related to pain across the most populated regions worldwide was observed to change over time before and after the onset of the COVID-19 pandemic. As internet searches are a surrogate for public interest, we assume that our data are indicative of an increased incidence of pain after the onset of the COVID-19 pandemic. However, as these increased incidences vary across both geographical and anatomical locations, our data could potentially facilitate the development of specific strategies to support the most affected groups.

## Appendix

Multimedia Appendix 1 Supplementary table.
