# Supplementary material for: Google Trends for Pain Search Terms in the World’s Most Populated Regions Before and After the First Recorded COVID-19 Case: Infodemiological Study
Source: J Med Internet Res. 2021 Apr 22;23(4):e27214. doi: 10.2196/27214 (PMC8064706; doi:10.2196/27214)
Supplement: Multimedia Appendix 1 [file jmir_v23i4e27214_app1.docx]

# Supplement 1

| Country | Country Code (ISO 3166-1 alpha-2) | Population | Weight | First Covid-19 Case |
| --- | --- | --- | --- | --- |
| Albania | AL | 2,862,427 | 0.003481 | 12.03.2020 |
| Austria | AT | 8,858,775 | 0.010773 | 26.02.2020 |
| Bosnia and Herzegovina | BA | 3,300,998 | 0.004014 | 06.03.2020 |
| Belgium | BE | 11,455,519 | 0.013931 | 04.02.2020 |
| Bulgaria | BG | 7,000,039 | 0.008513 | 08.03.2020 |
| Belarus | BY | 9,452,409 | 0.011495 | 28.02.2020 |
| Switzerland | CH | 8,544,527 | 0.010391 | 26.02.2020 |
| Czechia | CZ | 10,649,800 | 0.012951 | 02.03.2020 |
| Germany | DE | 83,019,213 | 0.100963 | 28.01.2020 |
| Denmark | DK | 5,806,081 | 0.007061 | 27.02.2020 |
| Estonia | EE | 1,324,820 | 0.001611 | 28.02.2020 |
| Spain | ES | 46,937,060 | 0.057082 | 01.02.2020 |
| Finland | FI | 5,517,919 | 0.006710 | 30.01.2020 |
| France | FR | 67,012,883 | 0.081497 | 25.01.2020 |
| United Kingdom | GB | 66,647,112 | 0.081052 | 01.02.2020 |
| Greece | GR | 10,724,599 | 0.013042 | 27.02.2020 |
| Croatia | HR | 4,076,246 | 0.004957 | 26.02.2020 |
| Hungary | HU | 9,772,756 | 0.011885 | 05.03.2020 |
| Ireland | IE | 4,904,240 | 0.005964 | 01.03.2020 |
| Iceland | IS | 356,991 | 0.000434 | 29.02.2020 |
| Italy | IT | 60,359,546 | 0.073406 | 31.01.2020 |
| Liechtenstein | LI | 38,378 | 0.000046 | 05.03.2020 |
| Lithuania | LT | 2,794,184 | 0.003398 | 28.02.2020 |
| Luxembourg | LU | 613,894 | 0.000746 | 01.03.2020 |
| Latvia | LV | 1,919,968 | 0.002334 | 03.03.2020 |
| Moldova | MD | 4,043,258 | 0.004917 | 08.03.2020 |
| North Macedonia | MK | 2,077,132 | 0.002526 | 27.02.2020 |
| Malta | MT | 493,559 | 0.000600 | 07.03.2020 |
| Netherlands | NL | 17,282,163 | 0.021017 | 28.02.2020 |
| Norway | NO | 5,328,212 | 0.006479 | 27.02.2020 |
| Poland | PL | 37,972,812 | 0.046180 | 04.03.2020 |
| Portugal | PT | 10,276,617 | 0.012497 | 03.03.2020 |
| Romania | RO | 19,414,458 | 0.023610 | 27.02.2020 |
| Russia | RU | 145,872,260 | 0.177402 | 01.02.2020 |
| Sweden | SE | 10,230,185 | 0.012441 | 05.02.2020 |
| Slovenia | SI | 2,080,908 | 0.002530 | 05.03.2020 |
| Slovakia | SK | 5,450,421 | 0.006628 | 07.03.2020 |
| Turkey | TR | 82,003,882 | 0.099728 | 12.03.2020 |
| Ukraine | UA | 43,993,643 | 0.053502 | 04.03.2020 |
| Kosovo | XK | 1,798,506 | 0.002187 | 14.03.2020 |
|  |  |  |  |  |
| Europe |  | 822,268,400 | 1 |  |
